# Silencing of the von Willebrand factor gene in proatherothrombotic *APOE∗3-Leiden.CETP* transgenic mice

**DOI:** 10.1016/j.rpth.2025.102699

**Published:** 2025-02-06

**Authors:** Yvonne K. Jongejan, Richard J. Dirven, Elisa Schrader Echeverri, Anke J.L. de Jong, Amanda C.M. Pronk, Sander Kooijman, Patrick C.N. Rensen, James E. Dahlman, Jeroen C.J. Eikenboom, Bart J.M. van Vlijmen

**Affiliations:** 1 Division of Thrombosis and Hemostasis, Department of Internal Medicine, Einthoven Laboratory for Vascular and Regenerative Medicine, Leiden University Medical Center, Leiden, Netherlands; 2 Wallace H. Coulter Department of Biomedical Engineering, Georgia Institute of Technology and Emory University School of Medicine, Atlanta, Georgia, USA; 3 Division of Endocrinology, Department of Internal Medicine, Einthoven Laboratory for Vascular and Regenerative Medicine, Leiden University Medical Center, Leiden, Netherlands

**Keywords:** atherothrombosis, hypercholesterolemia, mouse model, siRNA, von Willebrand factor

## Abstract

**Background:**

Elevated von Willebrand factor (VWF) levels correlate with higher risk of atherosclerosis-related arterial thrombosis (atherothrombosis). Silencing the *VWF* gene via small-interfering RNAs (siRNAs) could mitigate this risk. Previous studies successfully delivered siRNA to the endothelium of healthy, wild-type (WT) mice using lipid nanoparticles (LNPs).

**Objectives:**

This study aimed to investigate whether the LNP-siRNA strategy could achieve endothelium-specific *Vwf*-silencing under diseased conditions of prolonged hypercholesterolemia and atherothrombosis-prone vasculature.

**Methods:**

Female transgenic mice expressing a variant of human *APOE∗3* (ie, *APOE∗3-Leiden*) and human cholesteryl ester transfer protein (*CETP*), fed a cholesterol-enriched diet for 18 weeks, received an intravenous injection of LNP-encapsulated siRNA targeting *Vwf* (si*Vwf*) or scrambled control siRNA at 1.5 mg siRNA/kg. For comparison, the same LNP-siRNAs were administered to young, chow-fed WT mice. Plasma VWF and *Vwf* mRNA levels were measured 96 hours after injection, with immunofluorescence analysis of lungs and heart aortic root to assess VWF protein expression.

**Results:**

*APOE∗3-Leiden.CETP* mice exhibited elevated plasma VWF levels compared with WT mice, alongside hypercholesterolemia and aortic atherosclerosis. si*Vwf* administration led to over 85% reduction in plasma VWF in both strains, with a strong reduction in lung *Vwf* mRNA and VWF protein in the pulmonary endothelium. Similarly, si*Vwf* treatment resulted in the virtual absence of VWF protein in the endothelial lining of the aortic root of both nondiseased (WT mice) and atherosclerotic (*APOE∗3-Leiden.CETP* mice) vessel walls.

**Conclusion:**

The LNP-siRNA targeting *Vwf* strongly reduced plasma and endothelial VWF in mice with hypercholesterolemia and advanced atherosclerosis, indicating feasibility to target endothelial VWF under proatherothrombotic conditions.

## Essentials

• Lowering of von Willebrand factor (VWF) could be a therapeutic approach for atherothrombosis.
• Atherosclerotic *APOE∗3-Leiden.CETP* mice are atherothrombosis prone and feature high VWF levels.
• Lipid nanoparticle–mediated endothelial siRNA treatment reduced plasma and local VWF levels.
• *Vwf*-silencing is feasible under conditions of hypercholesterolemia and advanced atherosclerosis.

## 1 Introduction

Elevated levels of the plasma glycoprotein von Willebrand factor (VWF) are associated with an increased risk of developing arterial thrombosis and atherothrombosis [[1], [2], [3]]. On the contrary, individuals with low VWF levels in plasma, such as patients experiencing the bleeding disorder von Willebrand disease, have shown to be protected of developing arterial thrombotic events [4,5]. The current gold standard therapeutics for both acute atherothrombotic events and secondary thrombosis prevention are antiplatelet drugs, such as aspirin, clopidogrel, and ticagrelor [6]. Although effective in most patients, approximately 20% to 30% of patients experience antiplatelet resistance, a phenomenon in which there is therapeutic failure due to decreased response to antiplatelet therapeutics [7,8]. In addition, the use of antiplatelet drugs can induce a bleeding risk [9]. This means that there remains a need for safer and more personalized treatment options.

Lowering of high VWF levels by blocking the translation of *VWF* mRNA into VWF protein could be an interesting therapeutic approach for atherothrombosis. Silencing of *VWF* can be achieved using small-interfering RNAs (siRNAs). siRNAs are short double-stranded RNA sequences that can bind to the *VWF* mRNA, and through RNA interference by the formation of the RISC complex, mRNA will be degraded and subsequent translation into VWF protein will be hindered [10]. Previously, we have shown a successful silencing approach where murine endothelial *Vwf* was inhibited using a specific siRNA targeted toward murine *Vwf* [11]. As VWF is mainly produced by endothelial cells, using a delivery vehicle for targeted delivery of the siRNA to the endothelial compartment is required. Encapsulating the siRNA targeting *Vwf* into endothelial-specific lipid nanoparticles (LNPs) has previously demonstrated to reduce endothelial VWF [11,12]. Although the LNP-siRNA approach was successful, the studies were performed in young normolipidemic wild-type (WT) mice that have low circulating cholesterol and a healthy vasculature. Silencing of *Vwf* under diseased conditions may be more challenging. Hypercholesterolemia, and/or activation or damage of the aged and diseased vessel wall, may hamper successful delivery to or processing of the LNP-siRNA in the endothelial compartment and subsequent silencing of *Vwf*.

In this study, we evaluated whether our LNP-siRNA strategy also permits endothelium-specific silencing of *Vwf* under conditions of hypercholesterolemia and a diseased vasculature prone to developing atherothrombosis. These conditions were achieved by using transgenic mice expressing both the human apolipoprotein E3 (*APOE∗3*) mutation *APOE∗3-Leiden* and human cholesteryl ester transfer protein (*CETP*), that is, *APOE∗3-Leiden.CETP* mice, fed a cholesterol-enriched semisynthetic diet. *APOE∗3-Leiden.CETP* mice share the same genetic C57BL/6J background as WT mice used in previous studies with our si*Vwf* but develop diet-induced hypercholesterolemia and subsequent atherosclerosis [[13], [14], [15]]. Furthermore, *APOE∗3-Leiden.CETP* mice are known to respond well to lipid-lowering and antiatherosclerotic therapeutics [14], while an imbalance in anticoagulation due to silencing of the anticoagulant protein C can trigger atherothrombosis development [16]. Due to the need of considerable cholesterol exposure to induce advanced atherosclerosis development, extended cholesterol diet-feeding is required, and thus, *APOE∗3-Leiden.CETP* mice should be relatively old at the time of siRNA injection (approximately 30 weeks) compared with the 5-week or 6-week-old mice used in our previous *Vwf*-silencing experiments [11]. In this study, we assessed siRNA-mediated silencing of endothelial *Vwf* under diseased and healthy conditions through comparison of atherosclerotic *APOE∗3-Leiden.CETP* mice with WT mice.

## 2 Methods

### 2.1 Animals

*APOE∗3-Leiden.CETP* transgenic mice (on a C57BL/6J (B6) background) were bred in-house and genotyped as previously described [13]. Female *APOE∗3-Leiden.CETP* mice, from the age of 11 to 12 weeks, were fed a semisynthetic 0.15% cholesterol-enriched diet (containing 15% cocoa butter; Diet T; Ssniff-Spezialdiäten GmbH) for 18 weeks to induce advanced atherosclerosis in the aortic root area [16]. As plasma cholesterol and triglyceride (TG) levels are predictors of the extent of atherosclerosis development [17], at 4, 8, and 13 weeks on diet feeding, total cholesterol (#10166588130; Roche Diagnostics), and TGs (#11489232216; Roche Diagnostics) levels were determined in 20.0 μL EDTA plasma that was isolated from whole blood from the tail vein. Only mice with total cholesterol levels above 7.5 mmol/L at all 3 measured time points were included for further experimentation. To ensure the quality of the LNP-siRNA batch and as a reference and positive control of a normal effect to siRNA-mediated *Vwf*-silencing 5-week-old WT mice (B6 background) were obtained from Charles River, France (mouse strain #000664; The Jackson Laboratory). These WT animals were fed a chow diet (Rat and Mouse No. 3 Breeding; SDS) during experimentation. All mice were housed in conventional cages with a 12:12 hour light:dark cycle with ad libitum access to food and water. The primary researcher was blinded to the treatment order and animal treatment history at killing until data analysis. Animals were randomized using RandoMice software [18]. Experimental setup was in accordance with the Institute for Laboratory Animal Research Guide for the Care and Use of Laboratory Animals and had received approval from the Ethical Review Board for Animal Experimentation of the Leiden University Medical Center, Leiden, Netherlands, and Central Authority for Scientific Procedures on Animals of the Netherlands.

### 2.2 siRNA formulation, delivery, and *in vivo* administration

The lead siRNA, si*Vwf* (Axolabs), designed to specifically target the *Vwf* allele, has previously shown to be a strong inhibitor of *Vwf* in 5-week or 6-week-old WT B6 mice and was referred to in that paper as si*Vwf.B6* [11]. As a negative control treatment, 1 group received a scrambled control siRNA, si*Control* (Axolabs). siRNA sequences, including chemical modifications can be found in Supplementary Table 1. For endothelial delivery, siRNAs were encapsulated in 7C1 LNPs, as previously described [12,19]. LNP-siRNAs were cooled during packing and shipping from Atlanta, USA, to Leiden, Netherlands, and stored at 4 °C until administration. To ensure stability, LNP-siRNAs were administered within 4 weeks after formulation. Mice were given a single intravenous injection into the tail vein with a dose of 1.5 mg siRNA per kg bodyweight.

### 2.3 Lung mRNA and plasma analyses

After 96 hours of siRNA injection, 5-week or 6-week-old WT and ∼30-week-old *APOE∗3-Leiden.CETP* mice were anesthetized with a single intraperitoneal injection of a mixture of ketamine (100 mg/kg; Ketalin; aa-vet), xylazine (10 mg/kg; Sedamun 20 mg/mL; Dechra), and atropine-sulphate (0.1 mg/kg; Teva) and whole blood was drawn from WT and *APOE∗3-Leiden.CETP* mice as described [11]. The animals were then killed, and lungs and heart were directly isolated. Subsequent plasma analyses by ELISA measuring VWF antigen levels and VWF multimer analysis; for factor (F)VIII, an adapted activated partial thromboplastin time; and determination of *Vwf* mRNA transcript levels (from the left lung lobules) were also performed as previously described [11]. Primers used to perform the qPCR are indicated in Supplementary Table 2. For the VWF multimer assay, plasma samples were separated under nonreducing conditions on 0.8% to 1.5% SeaKem HGT agarose gels (Lonza), and visualized by western blotting with rabbit anti-hVWF polyclonal antibody (A0082-4.1 g/L; DAKO). VWF antigen levels were used to ensure uniform sample concentrations upon loading on the agarose gels. Densitometry images were generated and intensity of multimer bands were calculated using ImageJ (version 1.54k; National Institutes of Health) as previously described [20].

### 2.4 Histologic and immunofluorescent staining of VWF in lungs and aortic root area

Right lung lobules and hearts, isolated from WT and *APOE∗3-Leiden.CETP* mice, were fixated overnight in 10% formalin followed by paraffin embedding, cross-sectioned (5.0 μm) for lung and serial sectioned (5.0 μm) throughout the aortic root area for the heart [21], deparaffinized, and rehydrated. Histologic staining of the hearts with hematoxylin and eosin was performed according to an in-house protocol and mounting was done with Pertex (Histolab). Immunofluorescent staining of VWF in the lungs and aortic root area of the heart were performed and imaged as described [11]. To limit variations, sectioning, staining, and processing, the lungs and hearts were performed in the same series and imaging (ZEISS Axio Scan.Z1 slide scanner with ZEN blue 3.6 software), and semiqualitative analysis (Fiji ImageJ version 1.53t) were performed using identical settings. Per mouse, 4 lung sections and 10 to 12 heart aortic root sections were stained and imaged.

### 2.5 Statistical analysis

Graphic illustrations were generated, and statistical analyses were performed using GraphPad Prism 9.3.1 (GraphPad Software). Data are presented as individual data points with the median. The Mann–Whitney *U* test was used to determine statistically significant differences between 2 experimental groups. *P* ≤ .05 was considered to be statistically significant.

## 3 Results

### 3.1 Lowering of elevated VWF levels in a hypercholesterolemic environment

Before LNP-siRNA administration, cholesterol-enriched diet-fed *APOE∗3-Leiden.CETP* transgenic mice (∼30 weeks old) featured high plasma cholesterol (median, 16.9 mmol/L; range, 9.1-23.5 mmol/L) and TG levels (median, 5.3 mmol/L; range, 3.1-8.7 mmol/L), as determined in EDTA plasma from tail vein whole blood, similar to previous reports for these transgenic mice [15,16]. Additionally, before LNP-siRNA injection, cholesterol-enriched diet-fed *APOE∗3-Leiden.CETP* mice featured high plasma VWF levels (median, 210%; range, 209%-304%), as determined in citrated plasma from vena cava whole blood. This is in line with plasma VWF levels reported in cholesterol diet-fed *APOE∗3-Leiden* (without the human *CETP* transgene) mice [15,22] and in 15-week or 16-week-old *APOE∗3-Leiden.CETP* mice after 4 weeks of cholesterol-enriched diet feeding (data not shown; median, 195%; range, 120%-248%). The WT mice featured normal VWF levels (median, 100%; range, 49%-130%).

**Figure 1:**
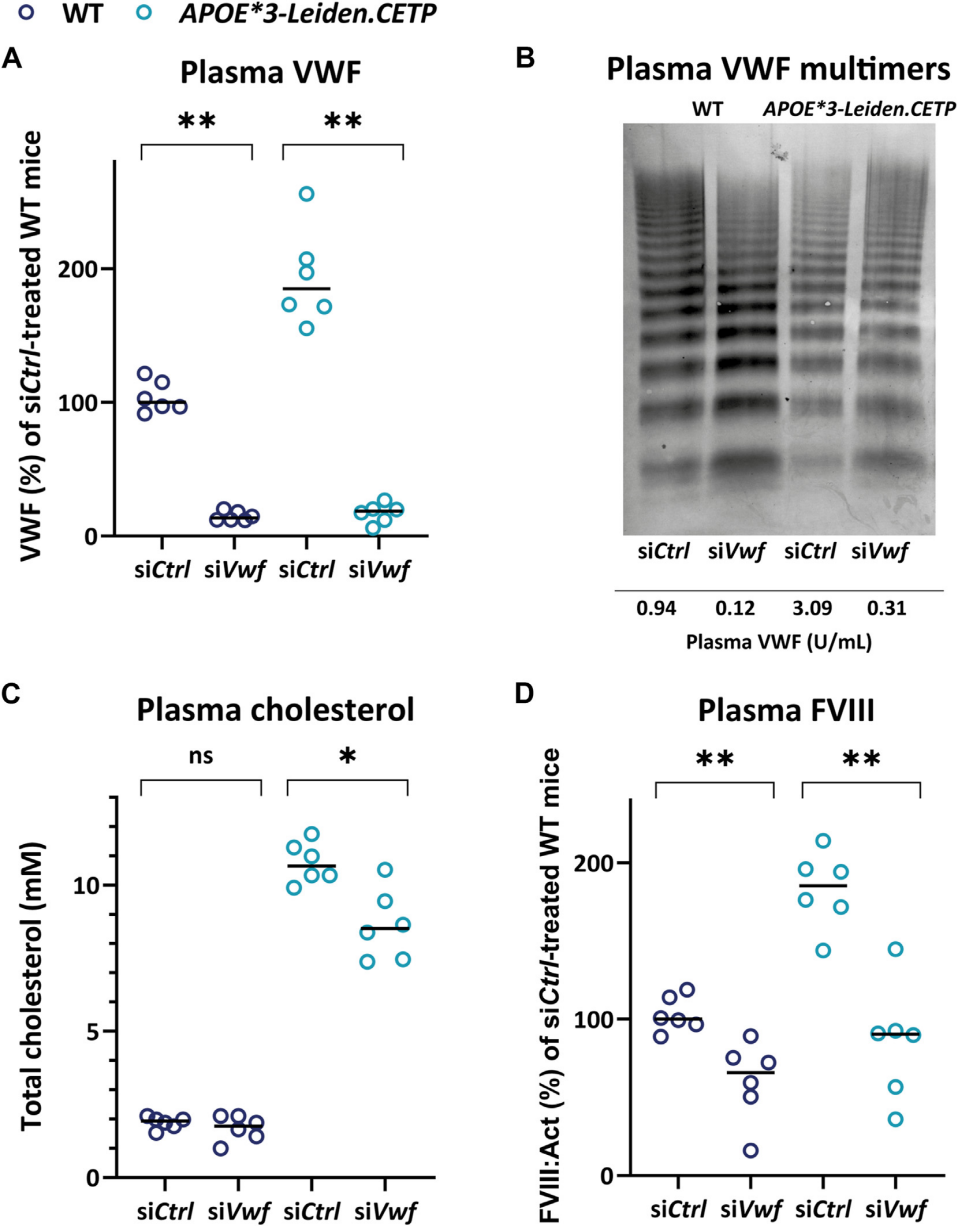
Effect of si*Vwf* treatment on plasma von Willebrand factor (VWF), cholesterol, and factor (F)VIII. (A) Plasma VWF levels of wild-type (WT; dark blue symbols) and *APOE∗3-Leiden.CETP* (turquoise symbols) mice that were treated with either si*Vwf* or its corresponding scrambled control si*Control*. (B) Representative VWF multimer pattern of plasma of a WT or *APOE∗3-Leiden.CETP* mouse treated with either si*Control* or si*Vwf*. Analysis was performed on citrated plasma and plasma of the si*Control*-treated WT mouse (left) was used as a reference for normal multimer patterns. Samples were diluted to a final sample concentration of 0.10 U/mL. Original sample concentrations were predetermined with ELISA, which could have resulted in the variable intensities. At the bottom of this panel, the plasma VWF levels are indicated for the individual mice that were used for the VWF multimeric pattern analysis. Note: To improve visibility of the multimer bands, the contrast of the entire image was increased. (C) Plasma cholesterol levels and (D) plasma FVIII of WT (dark blue symbols) and *APOE∗3-Leiden.CETP* (turquoise symbols) mice that were treated with either si*Vwf* or its corresponding scrambled control si*Control*. Values of the individual mice are shown with indication of the median value and were compared with the median of si*Control*-treated WT mice. Significant differences were determined based on comparisons between groups. ∗*P* ≤ .05; ∗∗*P* ≤ .01.

Following 18 weeks of cholesterol-enriched diet feeding and 96 hours post-siRNA administration, citrated plasma VWF levels remained high in the ∼30-week-old *APOE∗3-Leiden.CETP* mice after injection with si*Control* (Figure 1A). Similar to si*Vwf*-treated WT mice, which demonstrated reduced plasma VWF levels of a median of 87% (range, 80%-88%) in line with previous findings in si*Vwf*-treated WT mice of the same age [11,23], treatment with si*Vwf* in *APOE∗3-Leiden.CETP* mice resulted in a strong, and significant relative reduction of plasma VWF levels of a median of 90% (range, 86%-97%) compared with si*Control*-treated *APOE∗3-Leiden.CETP* mice (Figure 1A). This reduction resulted in low residual median plasma VWF levels (33.5 U/dL; range, 10.8-48.1 U/dL). Densitometry analysis on VWF multimers (Figure 1B) was performed to determine possible differences in ratios between small and large VWF multimers between treatment groups. Although visual inspection of the VWF multimers suggested an increase in small VWF multimers compared to si*Control* treatment, particularly in the WT mice, densitometry analysis did not show statistically significant differences (Supplementary Figure 1). So, despite the strong reduction at plasma level, the VWF multimer pattern was normal. This finding is in line with previous results observed for WT B6 mice treated with this B6-*Vwf* specific si*Vwf* [11]. The strong decrease in plasma VWF upon treatment with si*Vwf* coincided with a small but significant reduction in plasma cholesterol levels of a median of 10.6 mmol/L (range, 9.9-11.7 mmol/L) to 8.5 mmol/L (range, 7.4-10.5 mmol/L) in the *APOE∗3-Leiden.CETP* mice only (Figure 1C). Given VWF is the carrier protein for FVIII [24], as expected, treatment with si*Vwf* and consequent reduction of plasma VWF levels, also reduced plasma coagulation FVIII levels from a median of 100% (range, 88%-119%) to 66% (range, 16%-89%) in the WT mice and 185% (range, 143%-214%) to 90% (range, 36%-145%) in the *APOE∗3-Leiden.CETP* mice (Figure 1D), albeit that the reduction was not as strong as at the VWF level, which is in line with our previous findings in WT mice [11], and the feasibility to measure FVIII levels in *Vwf*^−/−^ mice [25].

### 3.2 Effect of *Vwf*-silencing on lungs and heart

**Figure 2:**
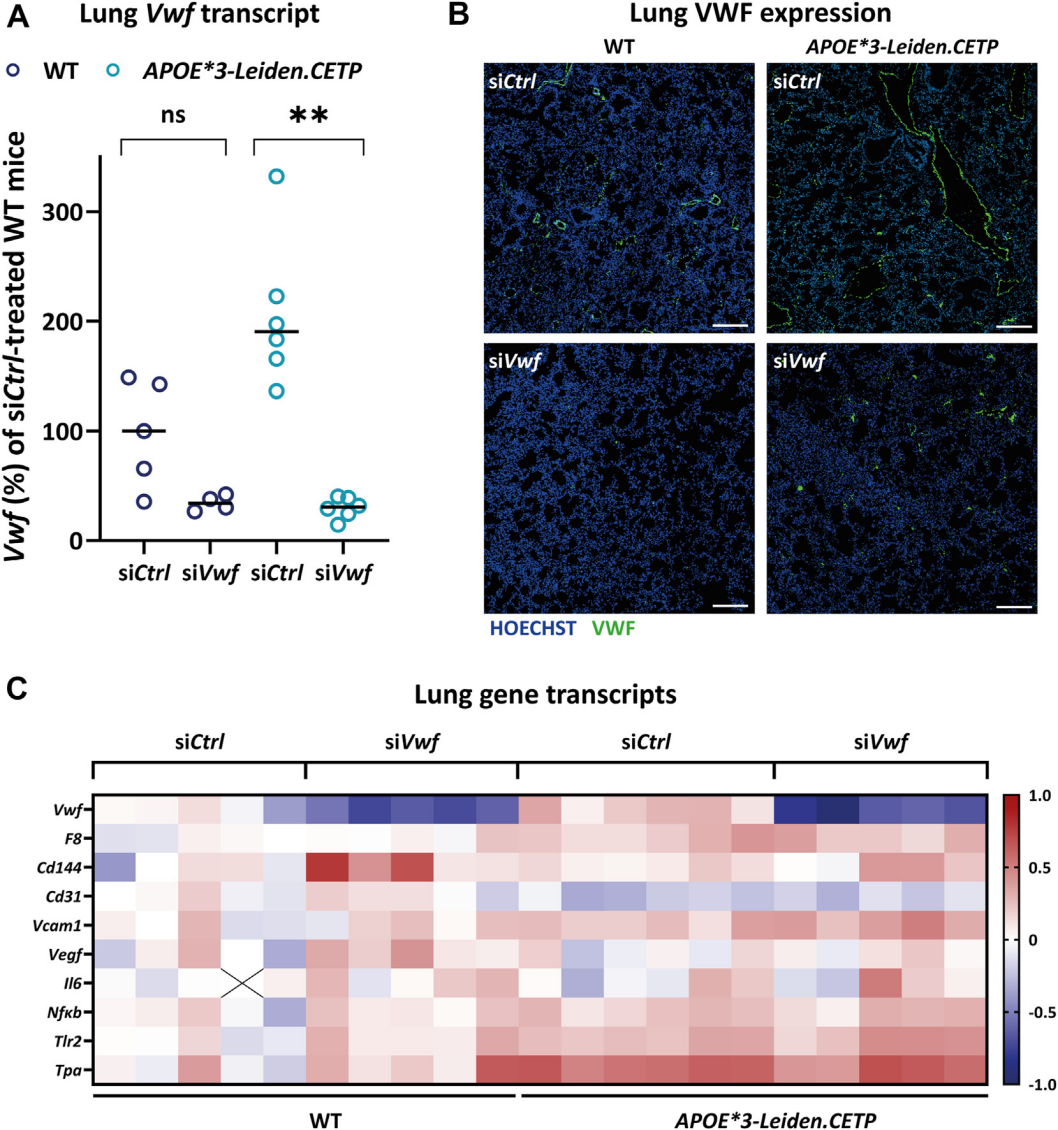
Effect of si*Vwf* treatment on lung *Vwf* expression. (A) Lung *Vwf* mRNA expression of wild-type (WT; dark blue symbols) and *APOE∗3-Leiden.CETP* (turquoise symbols) mice, treated with either si*Vwf* or its corresponding scrambled control si*Control*. Sample sizes per group were as follows: *n* = 5 si*Control*-treated WT mice, *n* = 6 si*Control*-treated *APOE∗3-Leiden.CETP* mice, *n* = 4 si*Vwf*-treated WT mice, and *n* = 6 si*Vwf*-treated *APOE∗3-Leiden.CETP* mice. Values of the individual mice are shown with indication of the median value and were compared with the median of si*Control*-treated WT mice. Significant differences were determined based on comparisons between groups. ∗*P* ≤ .05; ∗∗*P* ≤ .01. (B) Representative immunofluorescent images of von Willebrand factor (VWF) expression (green) of the lungs of si*Control* (si*Ctrl*)-treated or si*Vwf*-treated WT (left images) and *APOE∗3-Leiden.CETP* mice. Blue color represents nuclei (Hoechst staining). Scale bar is representative for 200 μm. (C) Lung mRNA transcript levels of genes associated with endothelial cells or inflammation. Data are presented as the log(2) of the fold change of an individual mouse compared with the median fold change per gene of all si*Control*-treated WT mice. No change in expression is indicated in white, downregulation compared with si*Control*-treated WT mice is indicated with blue, and upregulation with red.

*Vwf* transcript levels after siRNA treatment were determined in the lungs as this tissue is the major site of endothelial *Vwf* expression [26]. Similar to the findings for plasma VWF (Figure 1A), *Vwf* mRNA levels in the lung were higher in cholesterol-enriched diet-fed *APOE∗3-Leiden.CETP* mice when compared with chow-fed WT mice (Figure 2A). Treatment with si*Vwf* resulted in a strong and significant reduction of *Vwf* mRNA levels of 84% (range, 79%-92%) in the *APOE∗3-Leiden.CETP* mice. Immunofluorescent imaging of local VWF protein expression in the lungs further confirmed the inhibitory effect of si*Vwf* (Figure 2B). As expected, VWF-specific fluorescence was observed in the inner lining of the blood vessels in the lungs in the si*Control*-treated animals and decreased visibly in both si*Vwf*-treated WT and *APOE∗3-Leiden.CETP* mice, essentially following the observations in lung mRNA (Figure 2A) and previous findings in the lungs of WT mice [11]. To get a hint on whether *Vwf*-silencing also affects transcription of other endothelium-expressed genes in the lung, gene expression was measured of 5 endothelial and 4 inflammatory genes of which gene expression was suspected to be altered due to proatherogenic conditions (Figure 2C, Supplementary Figure 2) [27,28]. Higher expression of lung *Vwf* in the si*Control*-treated *APOE∗3-Leiden.CETP* mice coincided with higher gene expression of essentially all selected genes, apart from *Cd31*. The differences in gene expressions between the mouse strains likely reflect presence of endothelial activation upon extended exposure to high cholesterol, a characteristic also found in other murine atherosclerosis models, such as *Apoe*^−/−^ mice [29,30]. Interestingly, treatment with si*Vwf* solely affected expression of *Vwf* and not of any of the other genes in both WT and *APOE∗3-Leiden.CETP* mice. This included an unaffected expression of the *F8* gene, while plasma FVIII levels evidently were reduced upon treatment with si*Vwf* (Figure 1D), which is in concordance with literature indicating that reduced plasma FVIII levels rather result from increased FVIII clearance than reduced FVIII production [31,32].

**Figure 3:**
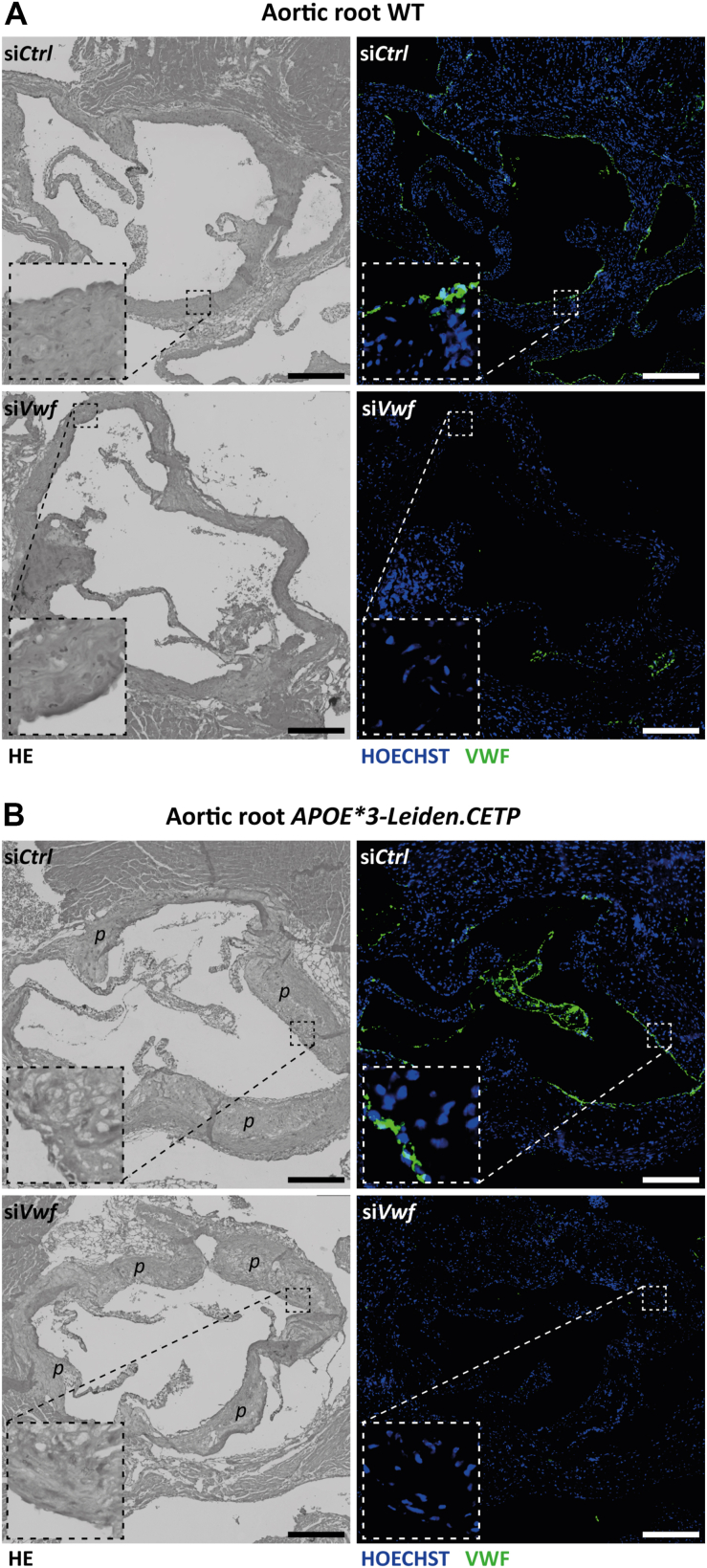
Effect of si*Vwf* treatment on the aortic root area of the heart. (A) On the left, representative hematoxylin and eosin (HE)–stained images of the aortic root area of the heart of wild-type (WT) mice treated with either si*Control* (si*Ctrl*) (upper image) or si*Vwf* (lower image). The right panels are representative immunofluorescent images of the aortic root of the same mouse. Blue, nuclei; green, von Willebrand factor (VWF) expression. (B) On the left, representative HE-stained images of the aortic root of the heart of *APOE∗3-Leiden.CETP* mice treated with either si*Control* (upper image) or si*Vwf* (lower image). Atherosclerotic plaques are indicated with *p*. The right panels are representative immunofluorescent images of the aortic root of the same mouse. Blue, nuclei; green, VWF expression. Between the HE and immunofluorescent sections of all mice is a 5.0-μm distance. Processing, imaging, and analysis of the images was performed using identical settings. Scale bar is representative for 200 μm; insets have a ×5 zoomed-in image of the selected area.

Next, the effect of *Vwf*-silencing was also assessed in the aortic root area of the heart, which is known to be the atherosclerosis and atherothrombosis-prone location in the *APOE∗3-Leiden.CETP* mice [16]. At the level of the aortic root area in the heart, cholesterol-enriched diet-fed *APOE∗3-Leiden.CETP* mice featured typical advanced atherosclerosis plaques characterized by the presence of foam cells, necrosis, fibrosis, and cholesterol crystals [33], which was absent in the WT mice (Figure 3, Supplementary Figure 3; hematoxylin and eosin–stained images). The luminal surface of the aortic root of si*Control*-treated WT animals stained clearly positive for VWF and followed the inner lining. In addition, the luminal surface of the advanced atherosclerotic lesions in si*Control*-treated *APOE∗3-Leiden.CETP* mice also stained positive for VWF. Similar to the results on the lungs and plasma, VWF staining was clearly visibly reduced after si*Vwf* treatment in the WT mice (Figure 3A). Despite the diseased state of the vasculature, treatment of si*Vwf* in the *APOE∗3-Leiden.CETP* mice (Figure 3B) also led to strongly reduced VWF staining. The limited number of animals included in the experiments (Figure 3, Supplementary Figure 3) and the 96-hour siRNA exposure was considered to be too small and too short to permit determination of the impact of si*Vwf* treatment on the extent of atherosclerosis. Nevertheless, this experimental setup seems sufficient to demonstrate that the LNP-si*Vwf* is capable to strongly silence endothelial *Vwf*, reducing circulating and localized VWF under conditions of hypercholesterolemia and a diseased atherothrombosis-prone vasculature.

## 4 Discussion

Reducing elevated circulating VWF levels could potentially lower the risk of developing atherothrombotic events. Using siRNAs targeting the murine *Vwf* gene, we previously showed a robust silencing of endothelial *Vwf* in normal WT mice with healthy vasculature [11]. In this mouse study, we have shown that siRNA-mediated robust silencing of plasma and endothelial VWF levels is also feasible in a setting of hypercholesterolemia and a diseased atherothrombosis-prone vasculature.

Cellular uptake of LNPs may involve interactions with endogenous apoE [34,35]. Especially for intrahepatocyte delivery, it is known that through the adsorption of apoE, the LNP can bind and internalize to the low-density lipoprotein receptors on the hepatocytes, facilitating uptake into the hepatocytes [[35], [36], [37]]. Hence, we anticipated that the hypercholesterolemia, and concomitant alterations in plasma proteins and apolipoproteins could interfere with the successful endothelial delivery of the siRNAs with the 7C1 LNP, observed under conditions of regularly circulating apoE in the WT mice. In addition, prolonged hypercholesterolemia conditions activate and alter the endothelium in a manner that might affect its capability to process the LNP and its siRNA content. Indeed, in this study, signs of transcriptional activation (Figure 2C) were observed in the lung endothelium of *APOE∗3-Leiden.CETP* mice. Nevertheless, in the hypercholesterolemic environment of the *APOE∗3-Leiden.CETP* mice, systemically administered LNP-siRNA could still reach the endothelial compartment and the RNA interference machinery to permit strong and effective endothelial RNA silencing, as here demonstrated for *Vwf.* This observation is in concordance with a previous study in cholesterol-fed atherosclerotic *Apoe*^*−*/*−*^ mice where endothelial delivery of the 7C1 LNP was demonstrated by means of visualization of a fluorescently labeled siRNA, and successful endothelial knockdown was demonstrated at the tissue transcript level (for the target gene transforming growth factor β) [38]. This study extends these findings to an atherosclerosis model with a more human-like lipoprotein profile, where apoE is still present, and demonstrating reduction at the protein level, both at the (diseased) endothelial lining of lung and aortic root and the circulating plasma pool.

Unfortunately, while the 7C1 LNPs are taken up successfully in the healthy [11,39] and diseased mouse vasculature as shown in this study, as well as in healthy nonhuman primates [19], therapeutic application in humans with this delivery vehicle is not feasible. Apart from the ionizable lipid 7C1, the LNPs only contain polyethylene glycol [12], which is required for stable LNP formulation [40]. However, polyethylene glycol makes the LNP nonbiodegradable. Biodegradability is needed to facilitate elimination of the LNP after siRNA delivery [41]. To avoid unwanted accumulation in the kidneys [12], optimization of the 7C1 LNP would be required for translational studies. This study encourages to reformulate the 7C1 LNP into a biodegradable LNP to make the next step in investigating the true antiatherothrombotic therapeutic potential of siRNA-mediated silencing of *VWF*. Future studies using siRNAs targeting human *VWF* encapsulated in a biodegradable LNP intravenously administered into a humanized *VWF* mouse crossed onto a proatherothrombotic *APOE∗3-Leiden.CETP* background would shine a light on this antiatherothrombotic potential and translatability of the approach.

Elevated plasma VWF levels have been associated with an increased atherothrombosis risk [1,2], while von Willebrand disease, characterized by low or defective VWF, is associated with a decreased risk of developing atherothrombosis [4,5]. Lowering plasma VWF levels would therefore be an interesting therapeutic target for mitigating atherothrombosis risk. The LNP-siRNA approach used in this study demonstrates the feasibility to strongly reduce total plasma VWF levels. A potent siRNA can also induce an overshoot in the silencing effect, potentially leading to an increased bleeding risk due to too low levels of VWF. One solution to prevent this overshoot effect would be to regulate dosing to maintain within the therapeutic VWF range; however, this would require frequent monitoring of plasma VWF levels. Another alternative would be allele-selective siRNAs. These allele-selective siRNAs are designed to target a single nucleotide difference between 2 alleles, resulting in silencing of only the targeted allele. Inhibition of only 1 of the *VWF* alleles result in a maximal 50% reduction in *VWF* mRNA and subsequent plasma VWF levels, which is considered to not lead to a relevant increase in bleeding risk. Previously, we demonstrated the feasibility of this approach to allele-selectively silence *Vwf* in an *in vivo* proof-of-concept study [11], where we targeted a single nucleotide difference in the *Vwf* gene between the alleles of the hybrid F1 offspring resulting from the cross between B6 and 129S1/SvImJ mice [23]. To use the allele-selective silencing approach for human *VWF*, siRNAs can be designed based on a genetic difference between alleles, for example, a disease-causing mutation. For our approach, however, targeting a mutation is no feasible as there are no mutations in the *VWF* gene to cause atherothrombosis [5,42]. Instead, common genetic differences that are not associated with causing disease, such as single nucleotide polymorphisms (SNPs), could be targeted. To achieve allele-selective inhibition, an individual should be heterozygous for such a SNP and the siRNA should be designed targeting that specific SNP. In the case of silencing of the *VWF* gene, targeting 1 of the 4 SNPs in the *VWF* gene with the highest minor allele frequency in the Caucasian population would target approximately 75% of this subpopulation [43,44]. This approach would require the development of 4 highly effective and allele-selective siRNAs, which would provide a safe approach with minimal bleeding risk.

In conclusion, our findings demonstrate the feasibility of using LNP-siRNAs to target endothelial VWF under proatherothrombotic conditions and pave the way for further studies of therapeutic silencing of *VWF* in vascular diseases.
