# Supplementary material for: Silencing of the von Willebrand factor gene in proatherothrombotic APOE∗3-Leiden.CETP transgenic mice
Source: Res Pract Thromb Haemost. 2025 Feb 6;9(1):102699. doi: 10.1016/j.rpth.2025.102699 (PMC11909755; doi:10.1016/j.rpth.2025.102699)
Supplement: Supplementary Material [file mmc1.docx]

**Supplemental information**

**Supplemental Table 1. siRNA sequences with their chemical modifications**

| **ID** | **Target** |  | **siRNA sequence** |
| --- | --- | --- | --- |
| si*Control* | - | scrambled si*Vwf* | 5'-dTAUAUCCAAUUAcFCCUAAcsa-3' |
| *siVwf* | B6 | c.1010T>C | 5'-dTGUACACAAAAUcUUCUCAcsa-3' |

^Note:^ Chemical modifications are indicated as follows: dT = DNA residue; G, C, A, U = RNA residue; g, c, a, u = 2’-O-Methyl modified residue; s = phosphorothioate backbone modification; underlined = position of the B6 strain-specific nucleotide.

**Supplemental Table 2. qPCR primer sequences**

| **Gene** | **Forward primer** | **Reverse primer** |
| --- | --- | --- |
| *Gapdh* | ACTCCCACTCTTCCACCTTC | CACCACCCTGTTGCTGTAG |
| *Vwf* | GCCTCAAGCAGAGCACAAAC | TCCTGCAGGCACAGGTAAAG |
| *F8* | CTTCACCTCCAGGGAAGGACTA | TCCACTTGCAACCATTGTTTTG |
| *Cd144* | CACTGCTTTGGGAGCCTTC | GGGGCAGCGATTCATTTTTCT |
| *Cd31* | CTCCAACAGAGCCAGCAGTA | GACCACTCCAATGACAACCA |
| *Vcam1* | TGTGGAAATGTGCCCGAAAC | GGTATTACCAAGGAAGATGCG |
| *Vegf* | CAGCACATAGGAGAGATGAGC | TCTTTCCGGTGAGAGGTCTG |
| *Il6* | CTGCAAGAGACTTCCATCCAG | AGTGGTATAGACAGGTCTGTTGG |
| *Nfκb* | AGCTGATGTGCATCGGCAAGTG | GTAGCTGCATGGAGACTCGAACAG |
| *Tlr2* | AAGATGCGCTTCCTGAATTTG | TCCAGCGTCTGAGGAATGC |
| *Tpa* | AAGTGGCTTGGGCAGAACATACAG | TCTTGGGCACATTGCTTGGA |

**Supplemental Figure 1. Effect of si*Vwf* treatment on the VWF multimeric structure in WT and *APOE*3-Leiden.CETP* mice. A** Representative multimeric pattern of WT (left) and *APOE*3-Leiden.CETP* (right) mice treated with either si*Control* or si*Vwf* (same image as Figure 1B). **B** Representative densitometric image and area under the curve (AUC, in pixels^2^) of si*Control*-treated WT mouse. The red lines represent the divisions from left to right in large, intermediate and small VWF multimers. The ratios in multimers within a treatment group are represented in the graph below. **C** Ratios of small (left graph) and large (right graph) multimers compared to the full multimer pattern per individual mouse. Values are presented with the median. Statistical analysis was performed using a non-parametric Mann-Whitney *U* test between si*Control* and si*Vwf* treatment groups within a strain but there were no significant differences.

**Supplemental Figure 2.** **Effect of si*Vwf* treatment on lung mRNA transcript levels of genes associated with endothelial cells or inflammation in WT and *APOE*3-Leiden.CETP* mice.** Bar charts of the endothelium-associated genes *F8, Cd144, Cd31, Vcam1* and *VEGF*, and inflammation-associated genes *Il6, Nfκb, Tlr2 and Tpa*, after treatment with either si*Control* or si*Vwf* in WT (blue) and *APOE*3-Leiden.CETP* (turquoise) mice. Significant differences were determined based on comparisons between groups. ns = not significant, *P* ≤ 0.05 = *.

**Supplemental Figure 3. Effect of si*Vwf* treatment on the aortic root of the heart in WT and *APOE*3-Leiden.CETP* mice.** HE stained (left, grey) and immunofluorescent stained (right, blue, and green) images of the aortic root area of the hearts of WT (left panel) and *APOE*3-Leiden.CETP* mice (right panel). The upper images include mice treated with si*Control* and the lower images include si*Vwf*-treated mice. Atherosclerotic plaques in the HE-stained images of the *APOE*3-Leiden.CETP* mice are indicated with *p*. Immunofluorescent images indicate nuclei in blue, and VWF expression in green. Between the HE and immunofluorescent sections of all mice is 5 μm distance. Scale bar is representative for 200 μm, insets have a 5x zoomed in image of the selected area. N=2 animals per strain per treatment. Note: The image of the second si*Control*-treated *APOE*3-Leiden.CETP* mouse includes an artefact that stained strongly positive for VWF, which based on other sections was identified as part of an aortic valve.

**Supplemental Figure 1**


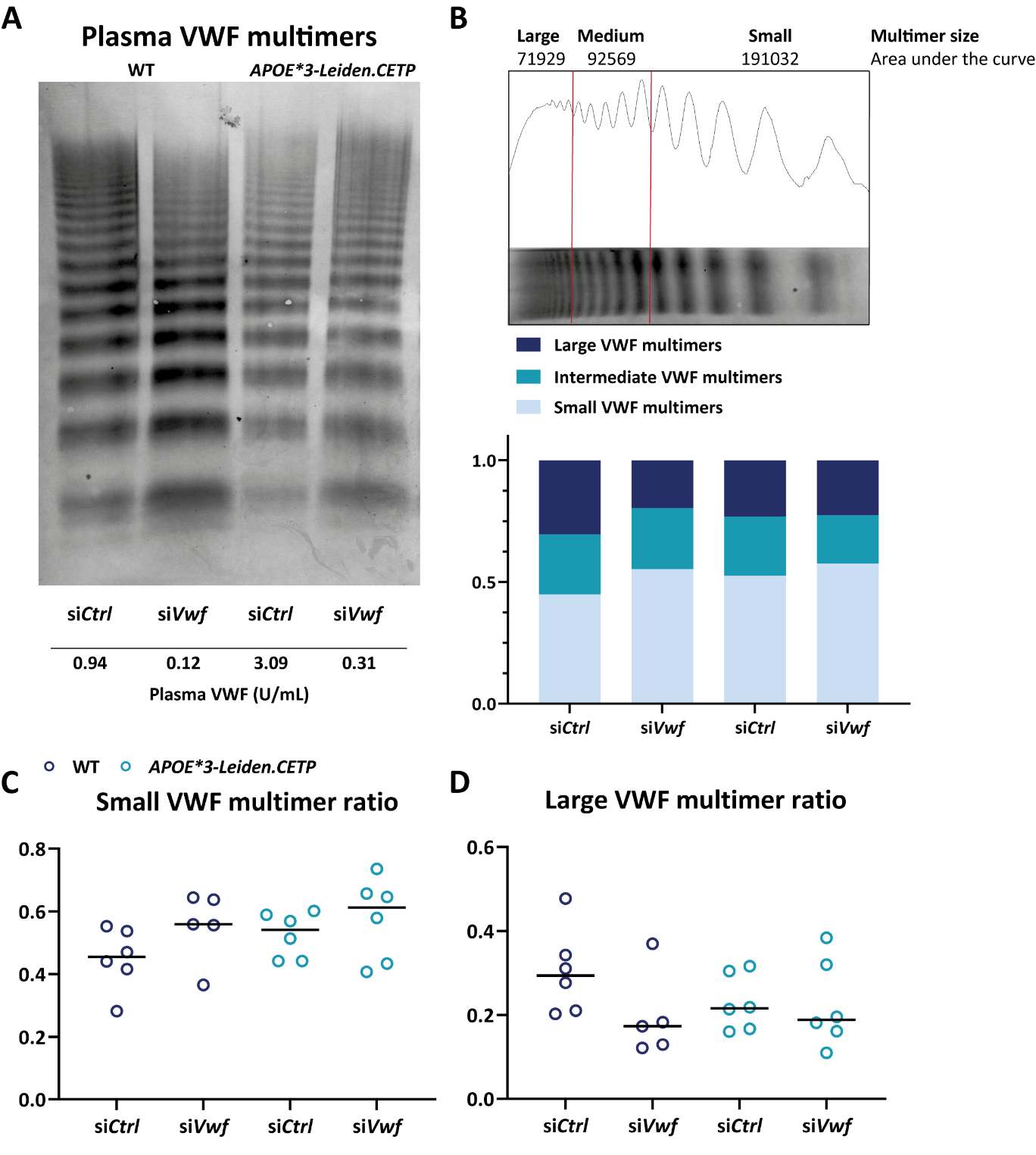


**Supplemental Figure 2**


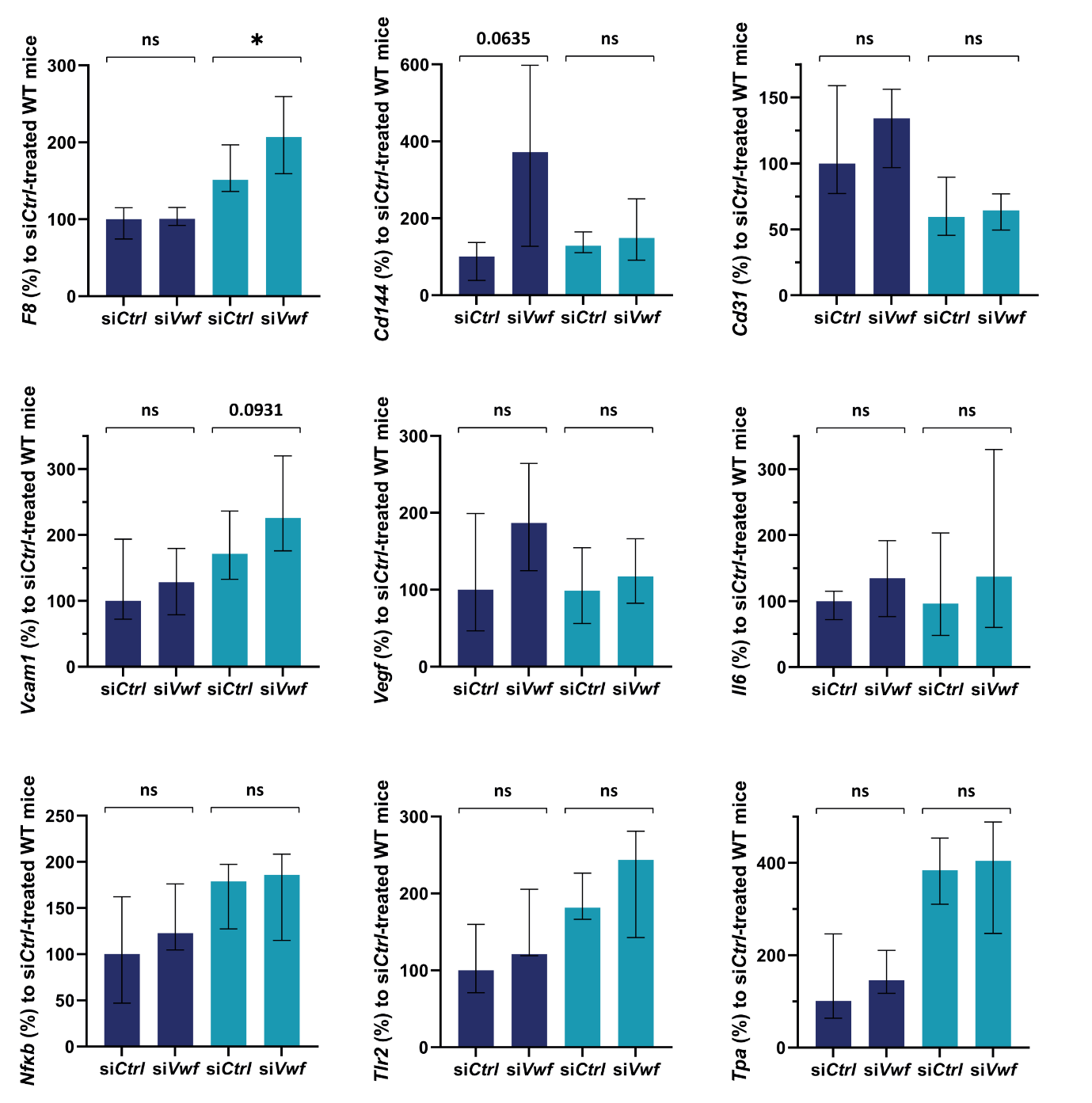


**Supplemental Figure 3**

**
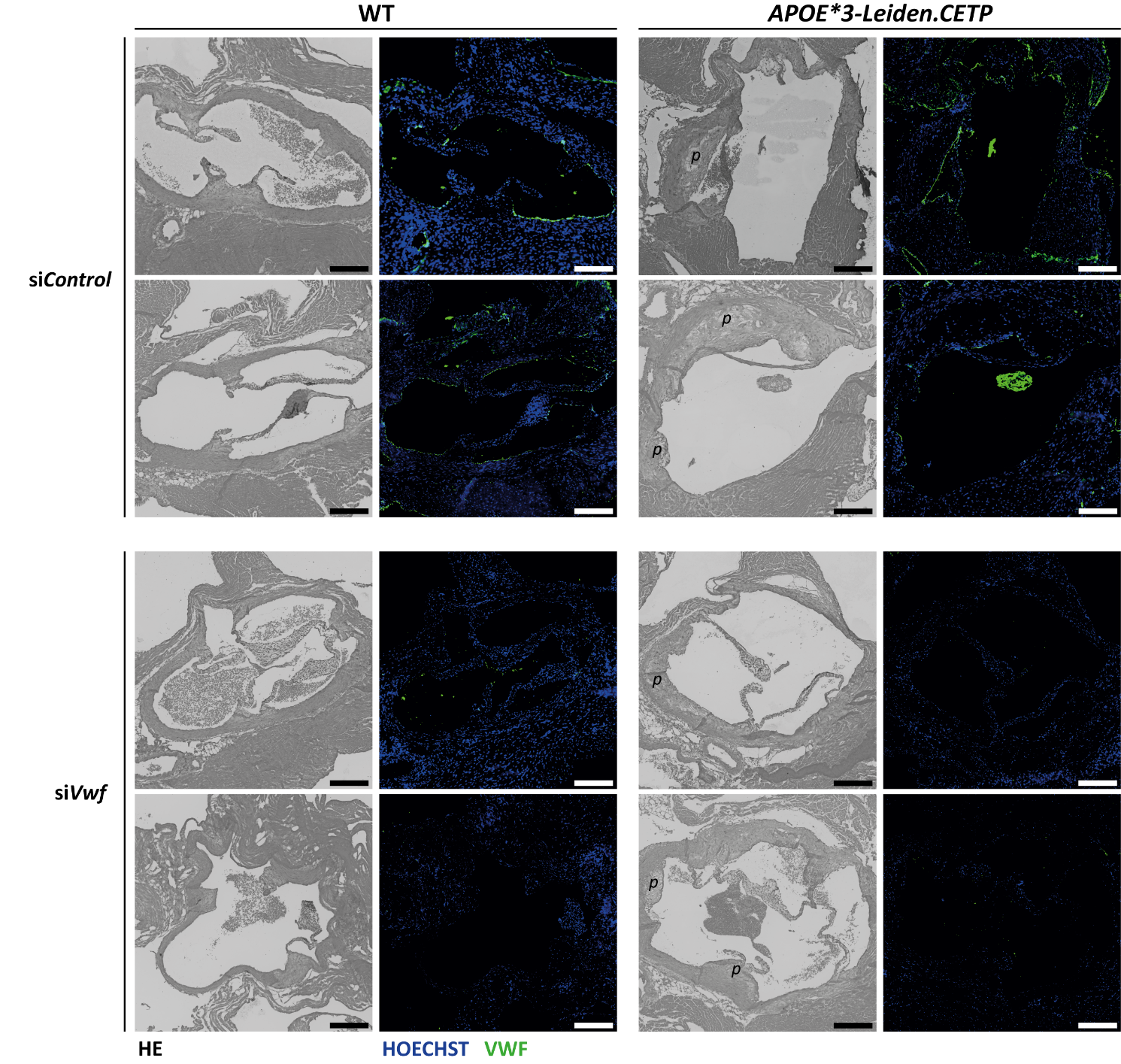
**
